# Generation of Dynamic Concentration Profile Using A Microfluidic Device Integrating Pneumatic Microvalves

**DOI:** 10.3390/bios12100868

**Published:** 2022-10-13

**Authors:** Chang Chen, Panpan Li, Tianruo Guo, Siyuan Chen, Dong Xu, Huaying Chen

**Affiliations:** 1 School of Mechanical Engineering and Automation, Harbin Institute of Technology, Shenzhen 518055, China; 2 School of Science, Harbin Institute of Technology, Shenzhen 518055, China; 3 Graduate School of Biomedical Engineering, The University of New South Wales, Sydney, NSW 2052, Australia

**Keywords:** concentration profile, dynamic, programmable, pneumatic microvalves, microfluidic, fluid resistance

## Abstract

Generating and maintaining the concentration dilutions of diffusible molecules in microchannels is critical for high-throughput chemical and biological analysis. Conventional serial network microfluidic technologies can generate high orders of arbitrary concentrations by a predefined microchannel network. However, a previous design requires a large occupancy area and is unable to dynamically generate different profiles in the same chip, limiting its applications. This study developed a microfluidic device enabling dynamic variations of both the concentration in the same channel and the concentration distribution in multiple channels by adjusting the flow resistance using programmable pneumatic microvalves. The key component (the pneumatic microvalve) allowed dynamic adjustment of the concentration profile but occupied a tiny space. Additionally, a Matlab program was developed to calculate the flow rates and flow resistance of various sections of the device, which provided theoretical guidance for dimension design. *In silico* investigations were conducted to evaluate the microvalve deformation with widths from 100 to 300 µm and membrane thicknesses of 20 and 30 µm under the activation pressures between 0 and 2000 mbar. The flow resistance of the deformed valve was studied both numerically and experimentally and an empirical model for valve flow resistance with the form of $R_{h}=ae^{bP}$ was proposed. Afterward, the fluid flow in the valve region was characterized using Micro PIV to further demonstrate the adjustment mechanism of the flow resistance. Then, the herringbone structures were employed for fast mixing to allow both quick variation of concentration and minor space usage of the channel network. Finally, an empirical formula-supported computational program was developed to provide the activation pressures required for the specific concentration profile. Both linear ($C_{k}$ = −0.2*k* + 1) and nonlinear $(C_{k}$ = ${\left(\frac{1}{\sqrt{10}}\right)}^{k})$ concentration distribution in four channels were varied using the same device by adjusting microvalves. The device demonstrated the capability to control the concentration profile dynamically in a small space, offering superior application potentials in analytical chemistry, drug screening, and cell biology research.

## 1. Introduction

Microfluidic technologies have been widely explored to create microenvironments with a concentration profile due to their prodigious advantages in automation, integration, and control of samples with extremely tiny volumes [1,2,3,4,5,6]. Existing microfluidic devices generate the concentration profile based on either passive self-diffusion [7,8,9,10], or continuous-flow mixing networks [11,12,13] before the observation region. The passive self-diffusion devices usually utilize diffusion across two parallelly laminar flows with chemical concentration discrepancy to generate a local concentration gradient [14,15,16]. To stabilize subjects, for example, cells in the middle observation chamber, the low-height channel [17,18], the membrane [19] and gels [20,21] were employed to form the obstacles. The unique advantage of high integration makes it possible for microfluidics to contain an array of chambers for high-through gradient analysis using limited samples [22,23,24,25]. However, the self-diffusion-based approach can only maintain a simple gradually decreasing concentration between a chemical source and a sink channel.

For more complex concentration profiles, the device requires intricate channel design such as proportional, pyramidal, and serial networks [26]. A proportional network controls the volumetric proportions of two streams by adjusting the flow resistance ratios of the channels before mixing [27,28]. However, the mixing ratio in a proportional network approach is limited by the channel design, making it hard to achieve dynamic adjustment. In contrast, the other two networks are more flexible. The pyramidal network repeats the splitting, mixing, and recombination of fluid streams to generate the concentration gradients. Since the concentrations are developed before the permutation combination, the pyramidal multistage-dilution device can produce more diverse concentration profiles [29]. For example, the Christmas tree network was designed to generate fixed gradient profiles by integrating multiple T-shaped mixing grades [11,30]. This approach has been extended to generate parabola [31], inverse proportion, cube root function [32], exponent [33], and linear [34] concentration variation across the width of the downstream observation channel. The concentration profiles were able to be dynamically tuned by adjusting the inlet flow rates [29,35]. Nevertheless, such networks are limited to a dilution range with few orders of magnitude, which confines the applications for dose–response experiments or high-throughput drug screening and optimization.

Comparably, the serial network, comprising a series of stepwise dilutions to mimic conventional manual serial dilutions, can generate logarithmic concentrations [36,37,38]. However, multiple serial dilution steps usually result in long flow channels, which require a large occupancy area. Additionally, the output concentrations profile highly relies on the network design, the majority of applications that use such networks failed to dynamically generate different profiles in the same chip. Since high order and dynamic adjustment are the two major requirements of the chemical environment in comparative experiments [39], there is a strong unmet need for modulating the arbitrary large range concentration profile dynamically in a diversity of experimental conditions. Thus, it is important to design a device empowering the serial network with dynamic regulation capability.

Since the pioneering report in 2000 [40], pneumatic microvalves have been extensively explored due to the overwhelming advantages of high integration [41], precision [42], and compatibility with PDMS-based devices [43]. They have been widely applied for multiple purposes including on/off switching [44] or even hydrodynamic trap [43,45]. Their key advantage is real-time adjustability. Moreover, manipulating microvalves can change the local flow resistance. Consequently, integrating the serial network device and pneumatic microvalves can be a promising approach for dynamically adjusting concentration profiles by precisely regulating the mixing ratio.

This paper proposed a prototype microfluidic device to dynamically change the chemical concentration in each of the four branch channels using programmed microvalves. The influence of activation pressure on the valve deformation and the resulting flow resistance was studied both experimentally and numerically. The bypass manometry measurement technique and the empirical model for the flow resistance provided a deep understanding of the irregular cross-section channel study in microfluidics. Then, a Matlab program was developed to predict the required activation pressures for generating varied custom dilution profiles. The herringbone microstructures were utilized to decrease the channel length and the response time for dynamic concentration variation. Finally, both linear and nonlinear concentration profiles were achieved in four branch channels and swapped by adjusting the pressure combination on four valves. This paper demonstrated the dynamic adjustment of the arbitrary concentration profile by active components in one microfluidic platform. The advantage of the device in the programmable alteration of the concentration will possess high application potentials in both chemical and biological analysis including *in vitro* cell culture and cytotoxicity test, drug screening as well as immunoassay.

## 2. Materials and Methods

### 2.1. Design and Fabrication

**Figure 1:**
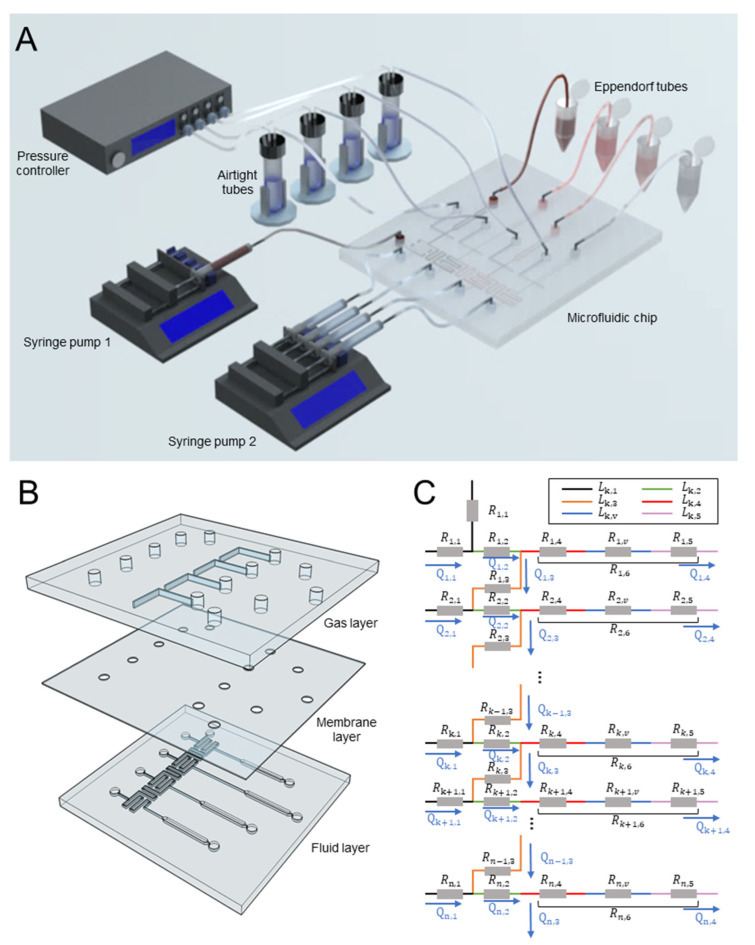
The microfluidic system for dynamic generation of concentration profiles in four parallel channels. (**A**) The schematic of the experimental platform consisting of a pressure controller, two syringe pumps, and a microfluidic. (**B**) The explosion view of the device with the gas, membrane, and fluid layers. (**C**) An equivalent circuit of the chip with n branch channels.

A microfluidic system (Figure 1A) was developed to dynamically regulate the chemical concentration in multiple branch channels by varying the pressure applied to the microvalves. The microfluidic system included a pressure controller, two syringe pumps, and a microfluidic chip (Figure S1). The chip was composed of a gas layer, a membrane layer, and a fluid layer from top to bottom (Figure 1B). There were four isolated gas channels (300 μm width and 50 μm depth) for individual control of the microvalves. The 20 μm thick membrane was deformed to modulate the cross-section (thus flow resistance) of the channel in the fluid layer after pressurization by the gas channels. The fluid layer had one chemical inlet, four inlets for diluting liquid (e.g., DI water), and four identical branch channels for concentrations generation. Specifically, there were five components: the inlet region (0.2 mm wide and 3 mm long), the mixing region with herringbone microstructures (0.2 mm wide and 20 mm long), the flow-shunting channel (0.2 mm wide and 20 mm long), the microvalve region (50 μm width and 300 μm length, with different flow resistance channels) and the observation region (1 mm width and 10 mm length).

The SU-8 molds were manufactured by standard lithography as described in the literature [41,45,46,47,48]. Briefly, after 30-min dehydration at 150 °C, two wafers were spin-coated with SU-8 2025 (MicroChem, Newton, MA, USA) at 1670 rpm to form 50 μm thick films. Following 3-min and 6-min baking at 65 and 95 °C, the wafers were exposed to UV light (URE-2000, Chinese Academy of Sciences, Beijing, China) through the photomask with the pattern of either gas channels or flow channels with a dose of 170 mJ/cm^2^. After the post-exposure baking at 65 and 95 °C for 1 and 6 min, the wafer with the flow-channel layer was spin-coated with SU-8 2025 at 1080 rpm to form a 75 μm thick film. After 3 min of 65 °C pre-bake and 9 min of 95 °C soft bake, the second photoresist layer was exposed to UV light with the dose of 210 mJ/cm^2^ through the mask with herringbone mixing structures and baked at 65 and 95 °C for 2 and 7 min, respectively. Finally, all wafers with photoresist films were developed in the SU-8 developer (MicroChem, Round Rock, TX, USA) to produce the molds of the gas and fluid layers.

Sylgard^®^ 184 elastomer base and curing agent (Dow Corning^®^ Corporation, Midland, TX, USA) were mixed with the weight ratio of 10:1 and completely degassed before being cast onto the SU-8 molds and baked at 70 °C for 2 h. Then, the Sylgard^®^ mixture with the curing ratio of 25:1 (base: curing agent) was spin-coated on the silicon wafer at 3750 rpm to form the 20 μm thick membrane before curing [45]. Afterward, the Polydimethylsiloxane (PDMS) replicas of the gas and fluid layers were peeled off the wafer. The inlet and outlet ports were manufactured using a puncher with an inner diameter of 0.75 mm. Three layers were then irreversibly bonded together after 30-second oxidation using oxygen plasma (PDC-002, Harrick Plasma, Ithaca, NY, USA). Finally, the inlet and outlet ports were connected to the tubing through 21-gauge, flat-end syringe needles. Before each experiment, the gas channel was filled with DI water to avoid activation pressure loss. To ascertain precise dimensions, the cross-sections of both membrane and microchannels were imaged using an inverted microscope (IX83 Olympus, Tokyo, Japan) equipped with a CCD camera (C11440-36U, Hamamatsu, Hamamatsu, Japan).

### 2.2. Concentration Prediction

A MATLAB-based customer program (Program 1) was developed to calculate the flow resistance in valve regions required for various concentration combinations in *n* (the branch number, *n* ≥ 2) parallel branch channels. The flow resistance was employed to guide the design of valve size. Figure 1C shows an equivalent circuit [26] of the chip. $L_{k,1}$ to $L_{k,3}$ and $R_{k,1}$ to $R_{k,3}$ represent the length as well as the flow resistance of the channel in front of the mixing region, the serpentine mixing channels before the microvalve and the channel between the *k*th microvalve and the (*k* + 1)th inlet, respectively. The flow resistance $R_{k,6}$ of the branch channel consisted of the flow resistance of the region between bifurcation and microvalve ($R_{k,4}$), the microvalve ($R_{k,v}$) and the observation regions ($R_{k,5}$).

To control the chip size, the dimensions of other components were fixed and listed in Table S1. Each observation region had an identical dimension (1 mm width and 10 mm length) and their flow resistance was referred to as $R_{k,5}$. The $L_{k,4}$ was determined as the minimum length required for the chemical concentration profiles, including $\sqrt{10}$ times decrease and linear decline with the slope of −0.2, in the four channels (from top to bottom). To generate the corresponding concentration profiles, the flow rates directed to the next mixing region ($Q_{k,3}$) were controlled by the required $R_{k,v}$, which were obtained by adjusting pneumatic valves. All detailed parameters in this design were listed in Table S1 in the Supporting Information (SI).

The flow resistance of the valve regions was calculated based on the desired concentration ratio. The concentration ratio between the *k*th observation channel and the chemical inlet was represented by $C_{k}$. As the flow rates in all dilution inlets were set to be a constant ($Q_{in}$), the concentration after mixing $(C_{k}$) depends on the split flow ($Q_{k,3}$), which was calculated by Equation (1):

(1) $$Q_{k,3}=\frac{Q_{in}\cdot C_{k+1}}{C_{k}-C_{k+1}}$$

The flow rate ($Q_{k,2}$) in the serpentine mixing region before the microvalve was the sum of a corresponding inlet flow ($Q_{in}$) and the flow ($Q_{k-1,3}$) from the former shunt (Equation (S1)). The flow rate in the observation region ($Q_{k,4}$) was the difference between $Q_{k,2}$ and $Q_{k,3}$ (the flow to the next level) (Equation (S2)). The flow resistance in all non-valve regions was calculated by Equation (S3). 

The flow resistance in the branch channels ($R_{k,6}$) can be given by Equation (2):

(2) $$R_{k,6}=\frac{R_{k,3}\cdot Q_{k,3}+R_{k+1,2}\cdot Q_{k+1,2}+R_{k+1,6}\cdot R_{k+1,6}}{Q_{k,6}}$$

Finally, the flow resistance in the microvalve region ($R_{k,v}$) can be calculated by Equation (3):

(3) $$R_{k,v}=R_{k,6}-R_{k,4}-R_{k,5}$$

The $R_{k,v}$ can provide guidance to microvalve dimension design and pressure manipulation both before and after chip manufacturing.

### 2.3. Simulation of The Valve Deformation and Flow Resistance

The deflection of the PDMS membrane was simulated to study its influence on the flow resistance across the valve using COMSOL Multiphysics 5.5 (COMSOL Inc.,Stockholm, Sweden). Membrane deflection was firstly simulated using a three-dimensional (3D) solid mechanics model (no fluid flow) consisting of a PDMS membrane with thicknesses (*t*) of 20 and 30 μm on a constant fluid channel (50 μm in both width and height) (see Figure S2A). The material details of the model can be found in Table S2. Briefly, the four vertical faces of the membrane were set to be fixed boundaries. A uniform pressure of 400 to 2000 mbar was applied on the top surface of the membrane. As reported in our previous paper [45], the contact boundaries between the vertical walls and the membrane were elastic support.

Based on the deflection results above, a second simulation was conducted to predict the flow resistance of the channel under the bending membrane. In the 3D model, the cross-section of semi-closed microchannels was built from the PDMS membrane deflection simulation (see Figure S2B), when the valve widths (*w*) varied from 100 to 300 μm. The 300 μm long channels (50 μm in both height and width) were set in front and behind the semi-closed microchannels. After applying a developed flow with a maximum velocity of 1.2 μm/s at the inlet of the whole channel, the flow rates and pressure drops in different semi-closed microvalve regions were obtained from the simulation. The relevant flow resistance ($R_{h}$) was calculated by Equation (4):

(4) $$R_{h}=k\frac{\Delta P}{Q}$$

where ΔP is the pressure drop between the inlet and outlet of the microvalve region and Q is the flow rate in the microchannel. *k* (see Equation (S6)) is the correction parameter to correct the error between simulation and experimental results.

### 2.4. Flow Resistance Measurement

A microfluidic chip with one straight flow channel and three pneumatic valves was employed to study the relationship between activation pressure and flow resistance. As shown in Figure S3, the bottom layer of the chip contained a straight fluid channel (50 μm width and 50 μm height) with two branches connecting a differential pressure sensor (DPS, Honeywell, Morris Plains, NJ, USA). The top layer consisted of three gas channels with the width of either 100, 200 or 300 μm and the height of 50 μm. The membrane between the gas layer and the flow layer was 20 μm thick. After device fabrication, the tubing connecting two ports of a pressure sensor was filled up with insulation oil (Yinglida, Shenzhen, China) to insulate the inner components and transfer the hydraulic pressure in microchannels [46]. The sensor was mounted on a sensor evaluation kit (Honeywell, Morris Plains, NJ, USA) with a microcontroller board (Arduino Uno Rev3, Somerville, MA, USA) to dynamically read the hydraulic pressure of the channel. 

The DI water was injected into the flow channel at 1 μL/min (Q) using a syringe pump (neMESYS 290N, CETONI, Korbussen, Germany). One of the three pneumatic valves was activated by the pressure ranging from 0 to 2000 mbar with an increment of 400 mbar using the pressure pump (Elveflow OB1 MK3+, Paris, France). The resulting pressure drop was recorded by the DPS when the membrane valve deflected at various pressure. The pressure drops (ΔP) were finally used for flow resistance calculation. The flow resistance of the valve activated at different pressure could be simply calculated by Equation (5):

(5) $$R_{h}=\frac{\Delta P}{Q}$$

### 2.5. Characterization of the Flow in Microvalves

The flow field under the valve region was characterized by the Micro-Particle Imaging Velocimetry system (Micro-PIV). Briefly, the 200 nm-diameter fluorescent particles (Huizhi Technology, Shanghai, China) were diluted in DI water with 0.1% Triton X-100. Under a flow rate of 1.3 μL/min, the pneumatic valve was controlled by activation pressures ranging from 0 to 2000 mbar with an increment of 400 mbar. A microscope (IX73, Olympus, Tokyo, Japan) integrating the double-pulse Nd-YAG laser (Vlite-135, Beamtech, San Francisco, CA, USA) was used for imaging. The excitation interval between the two lasers was set to 20 ms and the nanoparticles were recorded by a CCD camera (630091 PowerView 4MP-HS camera, TSI, Shoreview, MN, USA). The non-activated valves were imaged on the midplane, whereas the act-activated valves were imaged on the plane under the deformed membrane. Finally, the local fluid velocity was calculated by analyzing the displacement of the fluorescent particles.

### 2.6. Mixing Characterization

Standard herringbone microstructures were employed to enhance the mixing of fluids from different inlets. The mixing capability was visualized by the intensity of the fluorescein solution. The DI water and Fluorescein sodium (Energy Chemical Technology Co., Ltd. Shanghai, China) diluted in DI water (0.05%) were injected into the chemical inlet at 2.16 μL/min using syringe pumps. Both the mixing and the observation regions were imaged using a CCD camera with an exposure time of 10 ms. Finally, the fluorescence intensities in the mixing region on the micro photos were analyzed using ImageJ [49].

### 2.7. Generation of Dynamic Concentration and Data Analysis

Dynamically varied concentration was generated in four parallel channels by changing the pressure activating four pneumatic microvalves. Briefly, the fluorescein solution (0.05%) was injected into the chemical inlet at 0.46 μL/min or 4 μL/min for the nonlinear or linear profiles. The DI water was injected at 1 μL/min into the rest four inlets. The predefined pressures (see Table S3) acquired using the codes developed in Section 3.1 were applied to four microvalves to generate either linear decline or nonlinear increase concentration profiles. The fluorescence images in mixing regions were obtained via the CCD camera when the fluid flow became stable after the adjustment of the valve deflection. Finally, ImageJ was used to assess pixel intensity in the four mixing regions. Unless stated otherwise, the error bars in all figures represent the standard deviation (SD) of three replicates (N = 3).

## 3. Results and Discussion

### 3.1. Flow Resistance Calculation for Dynamic Concentration Profile

**Figure 2:**
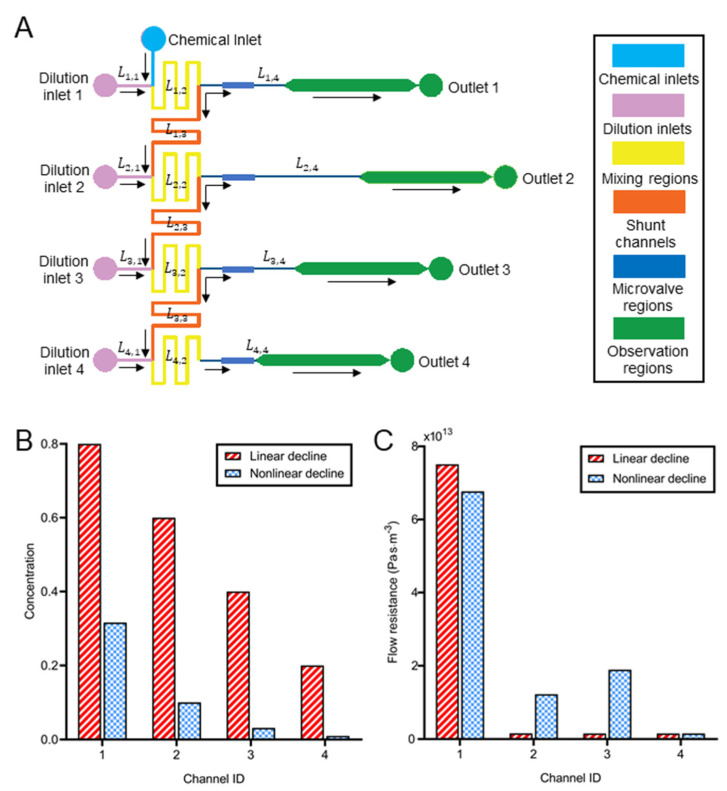
The calculation of the flow resistance for the dynamic concentration generation. (**A**) The schematic of the microchannel network in the flow layer. (**B**) The designed concentration in four parallel channels if the concentration at the chemical inlet was 1 (n = 4). The concentration in each channel was normalized to that in the first channel. (**C**) The flow resistance in the valve regions to acquire the concentration ratios in Figure (**B**).

A Matlab program (Program 1) was developed based on the equivalent circuit principle to calculate flow resistance in valve regions for distinct concentrations in the parallel branch channels. Since a four-branch chip was applied to the proof-of-concept experiments, the dimensions of four channels between bifurcation and microvalve ($L_{k,4}$, *k* = 1 to 4) were determined by the program to achieve the minimum flow resistance required for the concentration profiles (Figure 2A). Basically, $L_{k,4}$ were determined as the minimum length required for both linear ($C_{k}$ = −0.2*k* + 1) and nonlinear ($C_{k}$ = ${\left(\frac{1}{\sqrt{10}}\right)}^{k}$) chemical concentration profiles (Figure 2B). The corresponding flow resistance of these four valves is shown in Figure 2C and Table S4. 

The flow resistance in the first channel was significantly higher than the others. For example, to acquire a linearly declining concentration, the flow resistance in the first channel was 75.1 × 10^12^ Pa·s/m^3^, which was 48.14 to 48.77 times that in the rest three channels. Otherwise, most liquid might be directed to the first observation channel owing to the flow resistance distribution of the whole chip. Similarly, for the nonlinear concentration decline, the flow resistance in the first channels was 3.58 to 43.68 times that in the other channels. Moreover, the flow resistance in the second and third channels was 7.87 and 12.19 times bigger than in the last channel.

A similar calculation method and chip design had been applied for both linear, logarithmic or even six orders of magnitude concentration profiles generation in different devices [12,50]. Nevertheless, each device can only provide a constant profile. Active components such as pneumatic valves were introduced for dynamic control of ‘step-down’, ‘step-up’ or ‘gradient flip’ but the profiles were still constant [51]. The principle was the combination of two switchable pyramidal networks thus the changes were not arbitrary. To the best of our knowledge, this paper is the first demonstration of dynamic adjustment of the arbitrary concentration profile by active components in one serial network microfluidic platform.

### 3.2. Simulation of The Valve Deformation and Flow Resistance

**Figure 3:**
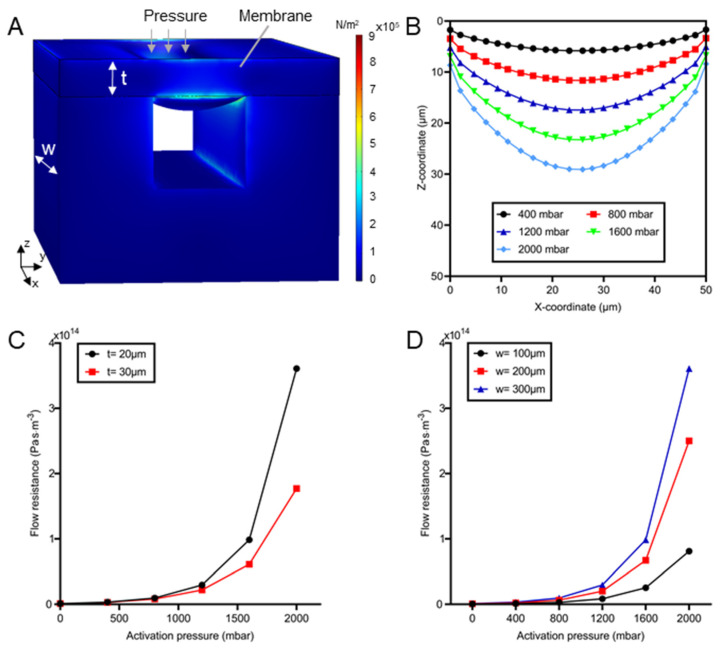
Study of the deformation and resulting flow resistance of pneumatic microvalves. (**A**) A 3D model of a microvalve. The width and the thickness of the membrane are represented by **w** and **t**, respectively. Color legend indicates the surface stress. (**B**) The cross-sectional profile of the membrane deflected at various pressure when **w** = 300 μm and **t** = 20 μm. (**C**) The flow resistance as a function of activation pressure for a valve with different membrane thicknesses and fixed width (300 μm). (**D**) The flow resistance as a function of activation pressure at different valve widths and a given membrane thickness (20 μm).

The key components of the concentration profile generator are the pneumatic microvalves, since they are employed to dynamically and independently tune the local flow resistance of each branch channel. Therefore, it is critical to study the deformation of the valve and the resulting flow resistance. The deflected membrane at 800 mbar is shown in Figure 3A. The membrane deflection increased as the augment of the activation pressure when *t* and *w* were 20 μm and 300 μm, respectively (Figure 3B). Although the membrane profile was parabolic, the maximum deflection had a linear relationship with the applied pressures as reported in our previous study [45]. The membrane deformed maximally for 5.8 µm (29.1 μm) and covered 9.2% (45.9%) of the channel cross-section when the activation pressure was 400 mbar (2000 mbar). Importantly, higher pressure formed the sharper corner between the vertical wall and the membrane (Figure 3A,B), which might greatly impact the flow resistance. 

The flow resistance was inversely related to the membrane thickness and proportional to the activation pressure (see Figure 3C). The thicker membrane resulted in smaller flow resistance at the same pressure. For instance, the resistance in the valve with a 20 μm thick membrane was 2.04 times that with a 30 μm-thick membrane when the pressure was 2000 mbar. That is because different deflections of the membrane with distinct thickness resulted in the flow resistance difference [45]. Whereas for the given membrane, the larger pressure might induce greater membrane deformation and significant raise in the flow resistance [52]. Figure 3C indicates that when the pressure increased from 400 to 2000 mbar, the resistance of the 20 μm-thick membrane dramatically rose by 109.4 times. This might be related to the difference in the cross-sectional shape of the channel. The channel cross-section with sharp edges (such as the triangle) normally has a higher flow resistance than that with a smooth boundary (such as the circle). Since the higher activation pressure resulted in a sharper corner between the membrane and channel walls, the flow resistance increased rapidly when larger pressure was applied.

Meanwhile, the flow resistance was also proportional to valve width (Figure 3D). Regardless of the valve widths, large pressure caused significant raise in flow resistance. When the pressure increased from 400 to 2000 mbar, the flow resistance increased from 1.06 × 10^12^ (3.27 × 10^12^) to 8.1 × 10^13^ (3.61 × 10^14^) Pa·s/m^3^ when the width was 100 μm (300 μm). When the width tripled from 100 to 300 μm, the resistance of the membrane under 400 mbar was raised by 2.08 times, whereas it dramatically increased 3.45 times when the pressure was 2000 mbar. Nevertheless, the flow resistance was not raised linearly with valve width. The phenomenon could be explained by the Equation (6) of flow resistance ($R_{h}$) for a rectangular channel in microfluidics [52]:

(6) $$R_{h}=\frac{12µL}{\left(1-0.63h/w\right)\cdot wh^{3}}$$

where µ is the dynamic viscosity of the fluid, *L*, *w* and *h* are the length, width and height of the rectangular microchannel, respectively. 

The valve width (*w*) in this paper was defined as the width of the gas channel, corresponding to the length of the liquid channel, which is *L* in Equation (6). Similarly, *w* in this equation corresponds to the channel width in the valve region (*w_v_*, see Table S1). Since the flow resistance is inversely proportional to the fourth power of height, the effect of height reduction due to membrane deflection was more pronounced than the growth of valve width. Simulation results indicated the deflection of a 20 μm-thick membrane under a 2000 mbar pressure initially rose with the increase in the microvalve width (Figure S4A) and then plateaued at approximately 150 μm (Figure S4B). As a result, the flow resistance may increase following the activation pressure with a rising slope when the valve width was smaller than 150 μm and be proportional to the valve width when it is bigger than 150 μm. 

The influence of pressure and membrane dimension (width and thickness) on the flow resistance was important for device design. It can be used to estimate the valve size required to generate predefined concentration ratios (see Equation (S3)). Previous studies calculated the flow resistance of the channel with the regular cross-section including circle, ellipse, and triangle [52]. However, the flow resistance of the pneumatic valve cannot be solved analytically, since the deflected membrane created a parabolic cross-section for fluid flow. To facilitate the control of flow resistance, valves enabling wider resistance ranges were preferred. Therefore, the valve with a 20 μm-thick membrane was chosen for the concentration profile generation in the following experiments. 

### 3.3. Flow Resistance Measurement

**Figure 4:**
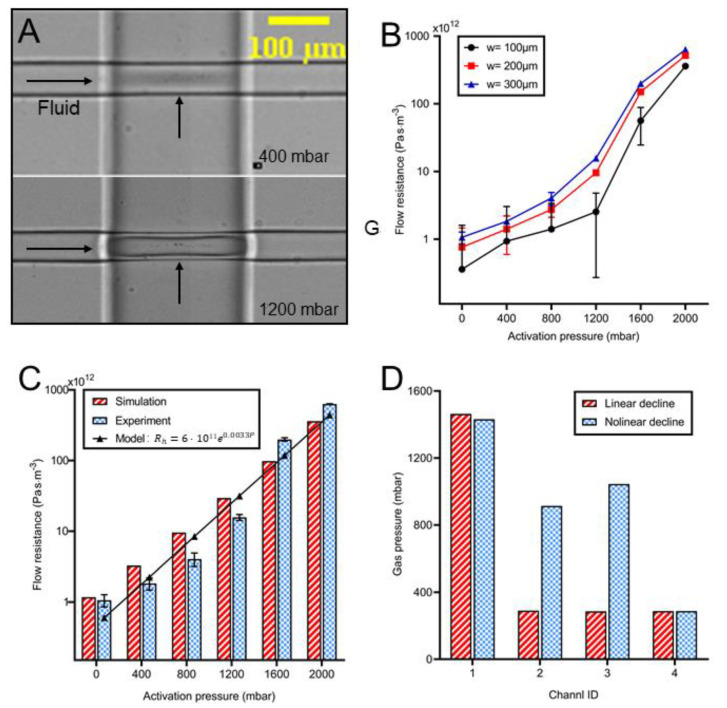
Measuring flow resistance in microvalves at various pressures. (**A**) The images of the deflecting membrane (20 μm thick) in a 200 μm long valve. (**B**) The flow resistance in the valve regions at various pressures. (**C**) The flow resistance (of a 300 μm valve) acquired either from experiment or simulation. The black line was a model describing the relationship between activation pressure and resistance (goodness: 0.86). (**D**) Activation pressures required to generate linear and nonlinear declining concentration profiles in four parallel channels (see Section 3.1).

The flow resistance in the microvalve region increased nonlinearly as a function of the activation pressure value. A custom system including a pressure sensor and a microfluidic chip (Figure S3) was developed to measure the flow resistance of the microvalve activated at various pressures. Figure 4A shows the deformations (top view) of the membranes in the 200 μm-wide microvalves activated at either 400 or 1200 mbar. As indicated by the shadow, the deflection under 1200 mbar was much larger than that under 400 mbar. 

Similar to the findings in Section 3.2 (Figure 3D), the flow resistance was proportional to both the activation pressure and the valve width (Figure 4B). Under the same pressure, the wider valve induced higher resistance. For the 100 μm valve, the flow resistance increased 386 times from 9.3 × 10^11^ to 3.6 × 10^14^ Pa·s/m^3^, as the activation pressure rose from 400 to 2000 mbar. However, for the 300 μm valve, the flow resistance enhanced 344.4 times from 1.83 × 10^12^ to 6.32 × 10^14^ Pa·s/m^3^, when the activation pressure varied in the same range. This suggested shorter valves were preferable if a significantly higher adjustable range of flow resistance was of interest. However, the standard deviation for shorter valves (100 µm) was much larger than that for long valves, indicating that higher accuracy of flow resistance was obtained for the long valve. Therefore, the 300 µm valves were used in the following experiments to generate a desirable dynamic concentration profile.

The flow resistance in a 300 µm valve acquired by either simulation or experiment was further compared in Figure 4C. Resistance from both methods had a difference of less than 57% for low activation pressure, whereas large errors (102% and 75%) occurred when 1600 and 2000 mbar were applied. This error may be related to the expansion of the gas channel, which has not been considered in the simulation. Since the walls of the gas channel were also formed by elastic PDMS, they may have a deformation under the pressure. Similar to the big wall collapse (8.6 μm for 2000 mbar) on the liquid channel (Figure 3A), there was a horizontal collapse on the gas channel, which was already observable under 1200 mbar pressure (Figure 4A). The pneumatic expansion of the gas channel under high pressure (more than 1200 mbar) prolonged the valve width, which significantly improved the experimental value of the flow resistance. An empirical model (*R^2^* = 0.86) was fitted to describe the relationship (Equation (7)) between the flow resistance ($R_{h}$) and the activation pressure (*P*):

(7) $$R_{h}=ae^{bP}$$

where *e* is the Euler number, *a* (6 × 10^11^) and *b* (0.0033) are constants. 

To the best of our knowledge, the flow resistance performance in the deforming microvalve with a non-regular cross-section has rarely been studied previously. Measurement of the flow resistance is essential for accurate generation of the concentration, owing to the error induced by the simplification in the simulation models and difficulty in the analytical calculation of the flow resistance in channels with irregular cross-section. The bypass manometry technique [53] was improved with a pressure sensor to accurately measure the pressure drop in this study. The measurement technique and the empirical model of the flow resistance and activation pressure may contribute to the microfluidic field.

Based on the model above, the required gas pressure for the desired concentration profile could be estimated at any predefined flow resistance. Take a four-branch channel chip as an example, the gas pressure on each microvalve for linear ($C_{k}$ = −0.2*k* + 1) and nonlinear ($C_{k}$ = ${\left(\frac{1}{\sqrt{10}}\right)}^{k}$) concentration profiles were calculated by Program 2 (see Figure 4D). Thus, the device integrated with active pneumatic microvalves can be altered to acquire the varied concentration profiles only in one chip which can achieve a spatial concentration dilution and minimize the manufactural error.

### 3.4. Characterization of The Flow in Microvalves

**Figure 5:**
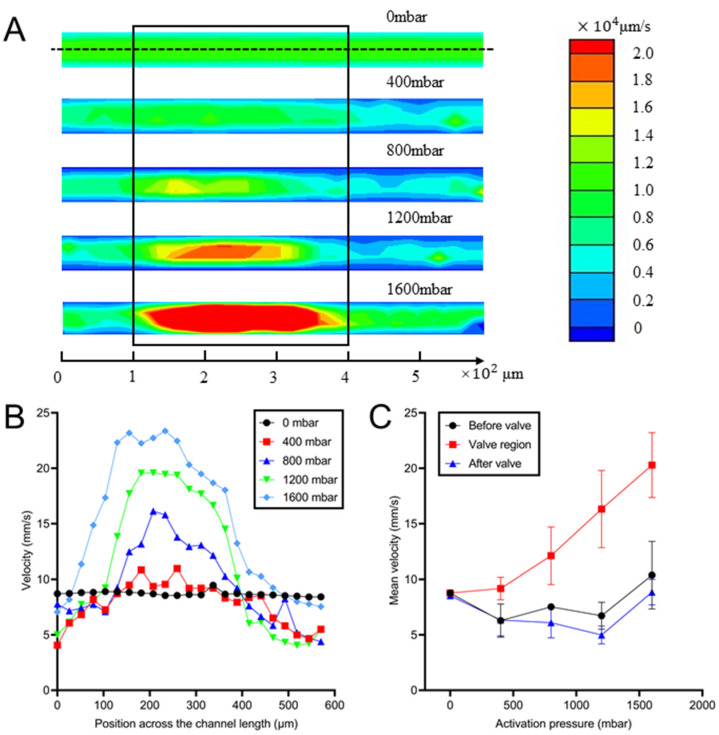
Characterization of flow in the valve zone. (**A**) The flow velocity contour in the valve region when the activation pressure was increased from 0 mbar to 1600 mbar. The black box represents the position of the valve. (**B**) Velocity distribution along the channel center under distinct activation pressures. (**C**) The mean velocity before, in and after the valve area at different activation pressures.

Micro-PIV was employed to measure the velocity under the 300 μm-width valve on the device mentioned in Section 3.3 and analyze the influence of the deformed membrane on flow resistance. When no pressure was applied to the valve, the velocity was uniform along the whole channel (see Figure 5A top). Nevertheless, there was a high-speed region under the membrane as the activation pressure on the valve increased from 400 to 1600 mbar (see Figure 5A), which was caused by the parabolic profile of the cross-sectional area (see Figure 3A). In addition, high-velocity regions expanded at higher activation pressure values, indicating a flatter membrane surface under higher pressure.

The velocity along the channel center (velocity distribution along the dotted line in Figure 5A) is shown in Figure 5B. Even though there was a low-speed layer on channel sides caused by the wall effect, flow velocity was uniform along the channel center line when no pressure was applied (see Figure 5B). However, under the activation pressure, there was a peak of speed in the valve region. That is because the flow rate was constant thus the local speed in the valve region, with a smaller cross-section, may be increased. In addition, the midplane (the velocity plane of no-pressure condition) had the highest speed and thus the no-pressured channel showed a higher speed in the no-valve region than that under an activation pressure. As the activation pressure increased from 400 to 1600 mbar, the peak velocity value rose from 10.96 to 23.36 mm/s. 

The comparison of the flow velocity between the valve region and other regions of the same channel is shown in Figure 5C. The velocity in the valve region was proportional to the activation pressure whereas there was an inverse relationship between the two factors for the channels outside of the valve when activation pressure was smaller than 1600 mbar. The mean velocity in the valve dramatically rose from 9.2 to 22.0 mm/s (Figure 5C) as the activation pressure increased from 400 to 1600 mbar. By contrast, the velocities outside of the valve were even smaller than the midplane velocity when the valves were not activated. As the 1600 mbar pressure was applied, the membrane was deformed to about 23 μm (see Figure 3B), which is around the midplane of the channel. As a consequence, the velocities outside the valve in this plane were similar to that at the midplane in no-pressure conditions. 

The experimental studies of flow fields when activation pressure was applied further explained the mechanism of flow resistance control. On the one hand, the deformed pneumatic valve may enhance the local speed as the flow rate was constant. On the other hand, the deformed valve was demonstrated to be less influential for the flow velocity profile out of the valve region. As a consequence, the activation pressure can only adjust the cross-section in the valve region and affect the local speed, without changing the flow rate in other regions. Since the flow variation outside the valve region was independent of activation pressure, it is indicated the addition of the valves may not affect fluid flow in this concentration profile generation device.

### 3.5. Mixing Characterization

**Figure 6:**
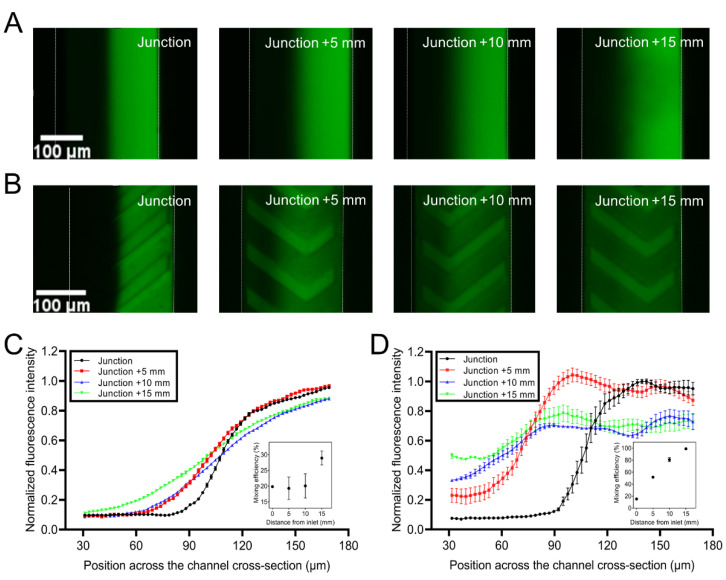
The mixing efficiency study. The images of the channels at different locations after dye and water were injected into devices (**A**) without and (**B**) with herringbone microstructures. The normalized fluorescence intensity based on the highest value of the fluorescent across the channels for the mixing characterization after flowing various distances in devices (**C**) without and (**D**) with herringbone microstructures. (Insets) Mixing efficiency after flowing varied distances in devices (**C**) without and (**D**) with herringbone microstructures. Error bars represent the standard error of the mean of five replicates.

To increase the throughput of mixed liquid, a higher inlet flow rate was required. However, at a high flow rate, the fluorescein was hardly mixed in the observation region, since the pure diffusion-based mixing required much longer channels. Therefore, herringbone structures were used to enhance mixing at a high inlet flow rate. In Figure 6A, the clear dark region in the vicinity of the fluorescence suggested uneven mixing between the dye and water at 15 mm away from the water-fluorescence junction when herringbone structures were absent. In contrast, the dye in the channel with herringbone structures was mixed only 5 mm away from the junction (Figure 6B), suggesting the efficiency of herringbone structures in mixing both liquids. The mixing characterization of the herringbone structures was analyzed quantitatively by measuring fluorescence intensity in the microchannel. In particular, the mixing efficiency (insets in Figure 6C,D) was calculated by dividing the mean intensity of the region from position 30 to 60 μm by 0.5. Figure 6C,D showed the fluorescence intensity on the lines (across the channel) 0, 5, 10 and 15 mm away from the merging point of the DI water and fluorescein in devices either with or without herringbone structures. In devices without herringbones (Figure 6C) there was only a 28.9% mixing after flowing for 15 mm. However, in devices with herringbone structures, the mixing efficiency reached 51.6% after 5 mm flowing and achieved 99.0% at the position 15 mm away from the mixing junction (see Figure 6D). The results indicated that the herringbone structures well enhanced the mixing in microchannel.

The performance of liquid mixing depends on both diffusion and convection. Most microfluidic devices generating concentration profiles employed pure diffusion across laminar flows. Therefore, the devices required long channels [54] and a low flow rate [36] to obtain steady concentration profiles. Plenty of technologies such as magnetic [55] or acoustic [56] have been exploited to enhance mixing efficiency [57,58]. Compared with complex active components, passive mixing can simplify both the device structure and the operation process. For instance, cylinder microstructures array [59] has been demonstrated to effectively enhance the mixing, utilizing just a 3 cm-length channel. Compared with the method of controlling fluid transformation sequences by cylinder microstructures, mixing with herringbone-inspired microstructures was able to overcome the diffusion limit in co-laminar microfluidic devices [60]. Previous evidence suggested that the scaling regime transited from a purely laminar regime to an entrance region turbulent regime with increasing Reynolds numbers [60]. Therefore, the herringbone microstructures overcome the limitation of pure diffusion and reduce the channel length for mixing [61]. As proved in Figure 6B, the herringbone microstructures induced the effective mixing in a 15 mm-length channel, which not only saved space but increased response speed for dynamic concentration variation.

### 3.6. Generation of Dynamic Concentration

**Figure 7:**
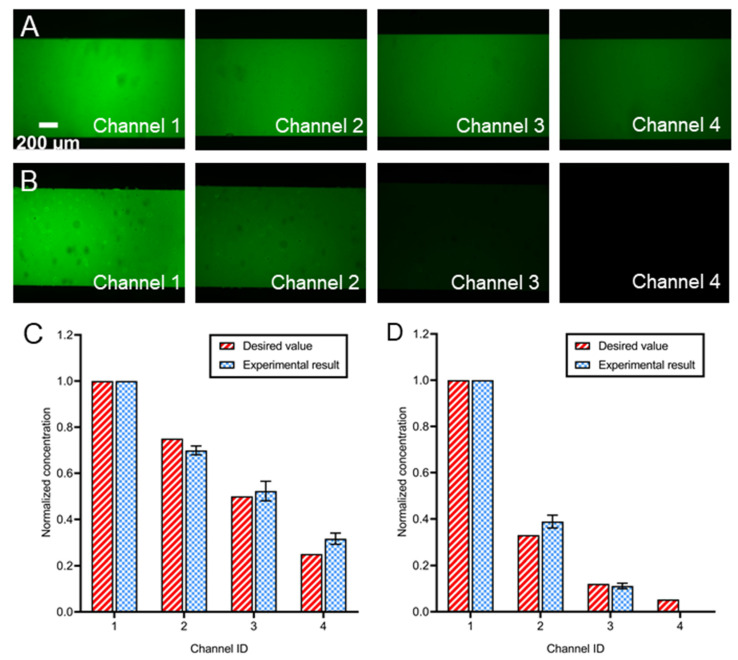
Generation of dynamic concentration using the microfluidic device. (**A**) Fluorescence images of the observation region with (**A**) linear and (**B**) nonlinear decline of concentration. The (**C**) linear or (**D**) nonlinear decline profile of the normalized concentration in four parallel channels. At least three different microchips were prepared and examined for each experimental condition.

The device’s capabilities in generating dynamically varying concentrations were validated by creating both linear ($C_{k}$ = −0.2*k* + 1) and nonlinear decline ($C_{k}$ = ${\left(\frac{1}{\sqrt{10}}\right)}^{k}$) concentration profiles. Figure 7A and B showed the fluorescent images of four observation zones 2 min after the activation of four valves (see Table S3 for the pressure combinations). The uniform fluorescence intensity demonstrated thorough and quick mixing since the images were taken only 2 min after valve activation. When the valve states combinations were swapped, the normalized concentration in four channels quickly varied between linear and nonlinear decline as shown in Figure 7C,D. Moreover, there was less than 26.6% difference in the normalized concentration acquired between the experiment and the desired value for both the nonlinear and the linear cases, except no signal was detected in Channel 4 for nonlinear profile, which may be because of the excitation limitation of the dye. The errors might be related to manufacturing accuracy or flow resistance calculation, as some structures such as herringbone parts were ignored in the calculation. 

However, these results suggested the ability of the proposed microfluidic networks in generating multiple concentration profiles in different channels. Limited by the serial dilution principle, it is difficult to generate the peak [11] or step-up [51] profiles such as those generated in the pyramidal network. Even though it is possible to acquire such profiles by switching some downstream dilution inlets to chemical inlets, the step-down profile demonstrated in this paper is enough for arbitrary concentration-dependent experiments. Previous studies generated temporal or spatial concentration profiles based on the serial dilution network [12]. However, long channels [37] or channels with low depth [50] were applied to provide a high-flow resistance network, which increased either the occupancy area or fabrication complexity. The pneumatic valves in this paper were able to generate a large range of flow resistance (variation in 594 times) within a small space (Section 3.3), which highly decreased the occupancy area. On the other hand, previous devices lack flexibility in the concentration variation, which is critical for comparative studies such as cell response experiments. Multiple components have been introduced in microfluidics for the fluid flow variation [62,63,64,65]. For concentration profile adjustment purposes, the active components include electronic ion pump [66], finger actuation button [67] and pinch [68] or pneumatic valve [69]. Among them, the pneumatic valve is ideal for dynamic control in microfluidics due to the excellence of accuracy, response time and integration [40,45,48]. In this paper, the addition of pneumatic enables the dynamic adjustment of flow resistance. As the result, the device was able to generate programmable arbitrary concentration profiles.

## 4. Conclusions

This paper reports a microfluidic platform that can dynamically generate the concentration profile in four channels by adjusting the pneumatic microvalves. A program was developed to provide the theoretical guidance for the design in dimension determination. Simulation results showed the deformation profile with varied activation pressures and valve dimensions. Afterwards, flow resistance under different conditions was studied by both numerical and experimental methods. An exponential rising tendency of flow resistance increasing by activation pressure was found. Moreover, local flow characterization in the valve region further demonstrated the flow resistance mechanism. Supported by the empirical formula from the experimental flow resistance study above, a computational program for predicting the activation pressure adjustment was developed to guide the concentration profile generator manipulation. To enhance the mixing efficiency, the herringbone structures were introduced and proved an efficient mixing at a 15 mm distance. Finally, a microfluidic device was demonstrated by generating both linear ($C_{k}$ = −0.2*k* + 1) and nonlinear ($C_{k}$ = ${\left(\frac{1}{\sqrt{10}}\right)}^{k}$) concentration profiles in the same chip with less than 26.6% error. In comparison to previous devices, the pneumatic valves are simple, reliable and easy to integrate and operate. The platform in this paper will be of great application potential in analytical chemistry, drug screening and cell biology research requiring concentration profile control.

## Data Availability

Not applicable.

## Supplementary Materials

The following supporting information can be downloaded at: https://www.mdpi.com/article/10.3390/bios12100868/s1, Figure S1: the photo of the experimental platform; Figure S2: the 3D models in the numerical study; Figure S3: the schematic of the microfluidic chip for flow resistance measurement; Figure S4: simulation results of the deflection of membranes with distinct valve widths; Table S1: details for four-branch design; Table S2: the parameters in simulation models; Table S3: the gas pressures required for different concentration profiles; Table S4: the flow resistance of microvalves for different concentration profiles; Program_1_Mixerdesign.m; Program_2_Pressurecalculator.m.
