# Supplementary material for: Generation of Dynamic Concentration Profile Using A Microfluidic Device Integrating Pneumatic Microvalves
Source: Biosensors (Basel). 2022 Oct 13;12(10):868. doi: 10.3390/bios12100868 (PMC9599525; doi:10.3390/bios12100868)
Supplement: Supplementary file 1 [file biosensors-12-00868-s001.zip › biosensors-1915905-supplementary.pdf]

biosensors

Supporting Information

Generation of Dynamic Concentration Profile Using A Microfluidic Device Integrating Pneumatic Microvalves

Chang Chen [1], Panpan Li [2], Tianruo Guo [3], Siyuan Chen [2], Dong Xu [2] and Huaying Chen [1,*]

[1] School of Mechanical Engineering and Automation, Harbin Institute of Technology, Shenzhen, Shenzhen 518055, China.
[2] School of Science, Harbin Institute of Technology, Shenzhen, Shenzhen 518055, China.
[3] Graduate School of Biomedical Engineering, The University of New South Wales, Sydney, NSW 2052, Australia.
* Correspondence: chenhuaying@hit.edu.cn

1. Materials and Methods

1.1. Design and Fabrication

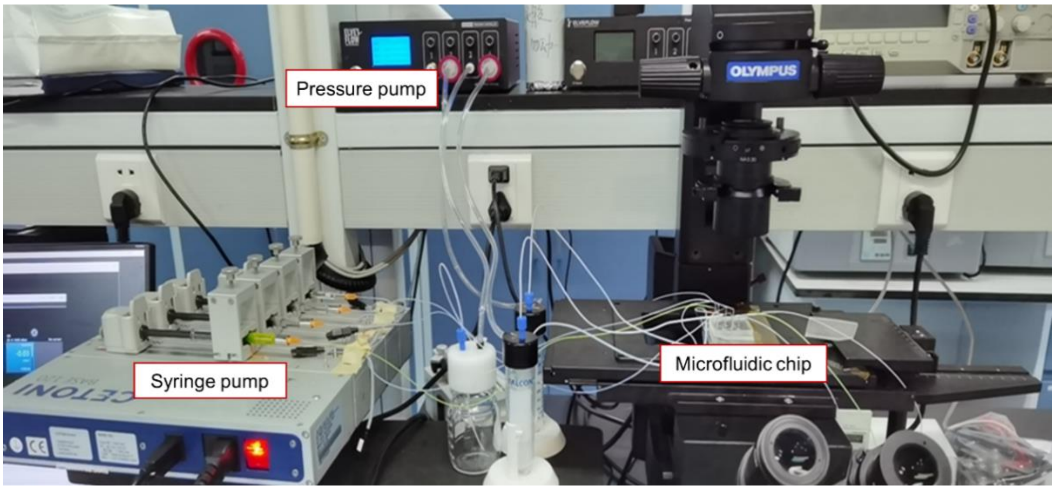

Figure S1. The photo of the experimental platform consisting of a pressure controller, syringe pumps, and a microfluidic device for dynamic concentration profile generation.

1.2. Concentration Prediction

Table S1. Details for four-branch design in Program 1.

Parameters	Symbols	Value/Range	Unit
Dynamic viscosity	μ	1.01 × 10^{-3}	[Pa·s]
Channel length before mixing region (*k*th stage)	$L_{k,1}$	3	[mm]
*k*th mixing region length	$L_{k,2}$	20	[mm]
Channel length between *k* and *k* + 1 stages	$L_{k,3}$	20	[mm]
1st outlet channel length	$L_{1,4}$	7.17	[mm]
2nd outlet channel length	$L_{2,4}$	13.6	[mm]
3rd outlet channel length	$L_{3,4}$	8.04	[mm]
4th outlet channel length	$L_{4,4}$	4.70	[mm]
*k*th microvalve region length	$L_{k,v}$	300	[μm]
*k*th concentration region length	$L_{k,5}$	10	[mm]
Channel width except valve and concentration region	w	200	[μm]
Microvalve region width	w_v	50	[μm]
Concentration region width	w_c	1	[mm]

The flow rate in the serpentine mixing region before microvalve $Q_{k,2}$ was the sum of a corresponding inlet flow and fluid from the former shunt:

$$Q_{k,2} = Q_{in} + Q_{k-1,3} \quad \text{(Equation S1)}$$

In the observation region, the flow rates were the remaining part after the flow from the serpentine mixing region divided into shunt region:

$$Q_{k,4} = Q_{k,2} - Q_{k,3} \quad \text{(Equation S2)}$$

The flow resistance in the non-valve region was calculated by traditional equation:

$$\begin{cases} R_{k,m} = \dfrac{8\mu L_m}{{r_m}^2 A_m}, & w = h \\ R_{k,m} = \dfrac{12\mu L_m}{w_m \cdot h^3 (1 - 0.63 w_m / h)}, & w > h \end{cases} \quad \text{(Equation S3)}$$

where μ is the viscosity of the fluid, L_m, w_m, h is the length, width, height of the *m*th microchannel, respectively, A_m is the cross-sectional area of the *m*th channel, and r_m is the hydraulic radius of the *m*th channel, which is given by:

$$r_m = \frac{2A_m}{C_m} \quad \text{(Equation S4)}$$

where C_m is the length of the perimeter of the *m*th channel.

1.3. Simulation of The Valve Deformation and Flow resistance

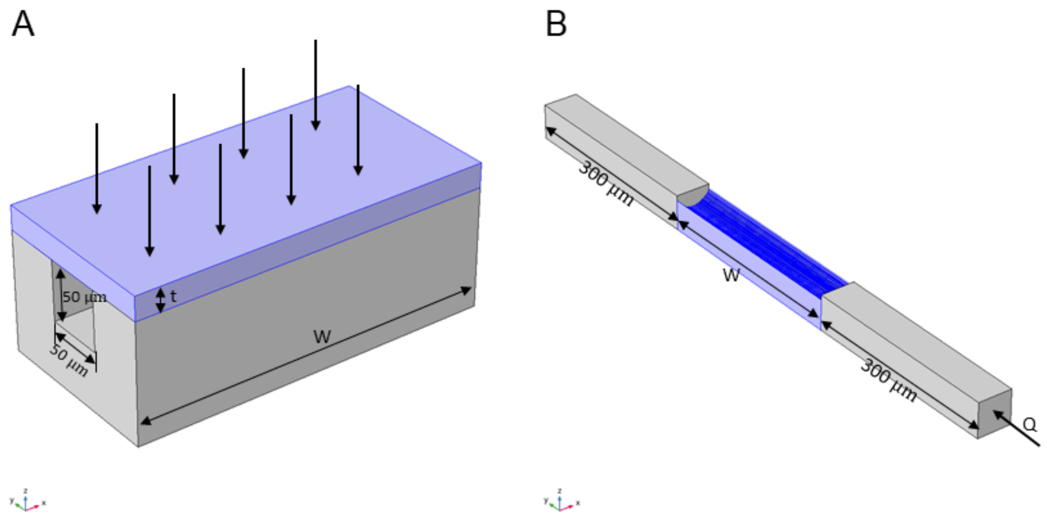

Figure S2. (**A**) The 3D model of the membrane-equipped channel for the numerical study of pneumatic valve deformation. The blue part represents the PDMS membrane. (**B**) The 3D model of the deformed microvalve region with the straight upstream and downstream channels for the numerical study of the flow resistance under varied activation pressures. The blue part represents the microvalve region.

Table S2. The parameters in simulation models.

Parameters	Symbols	Value/Range	Unit
The elastic modulus of membrane	$E1$	0.98	[MPa]
The elastic modulus of walls	$E2$	2.66	[MPa]
Poisson's ratio of the model	v	0.47	1

Since some errors from the manufacturing process or measurement may affect the values of the flow resistance measured in the experiment, we employed a correction parameter (k) between simulation and experimental results for the 300 µm-length valve with the 20 µm-thick membrane. It was calculated by:

$$k = \frac{R_{h,exp}}{R_{h,sim}} \quad \text{(Equation S5)}$$

where k is the correction parameter, $R_{h,exp}$ and $R_{h,sim}$ is the flow resistance from experiment and simulation, respectively.

Since the correction parameter for simulation was not constant and increases with the activation pressure, it was obtained from the fitting equation of:

$$k = 0.85 \cdot e^{0.002 \cdot P} \quad \text{(Equation S6)}$$

where e and P is Euler number and the activation pressure, respectively.

1.4. Flow Resistance Measurement

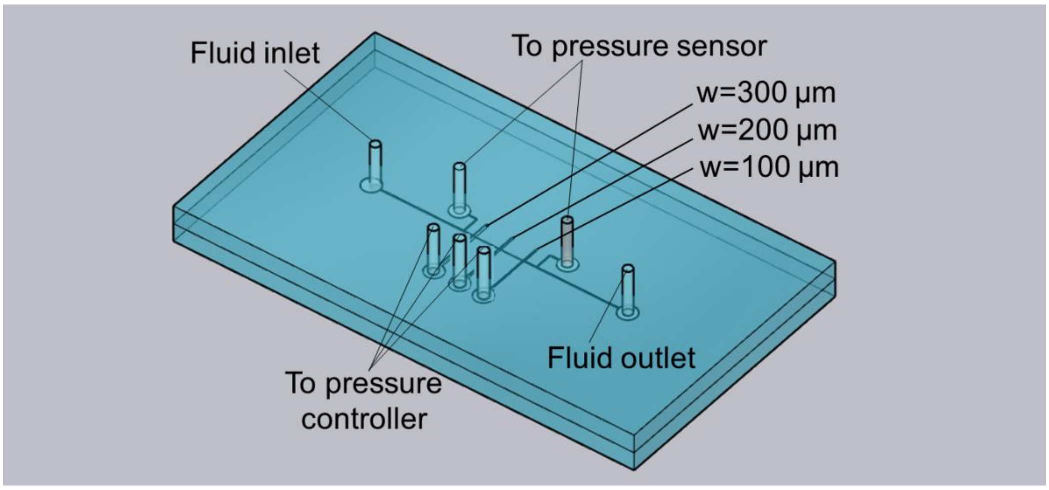

Figure S3. The schematic of the microfluidic chip consisting of three valves with the width of 100, 200 and 300 μm.

1.5. Generation of Dynamic Concentration and Data Analysis

Table S3. The gas pressures required for different concentration profiles.

Channel ID	Nonlinear Decline (mbar)	Linear Decline (mbar)
1	1431.98378	1463.501
2	913.9742647	289.9568
3	1044.874156	286.1225
4	287.8659362	287.8659

2. Results and Discussion

2.1. Flow Resistance Calculation for Dynamic Concentration Profile

Table S4. The flow resistance of microvalves for different concentration profiles.

Channel ID	Nonlinear Decline ($\times 10^{12}$ Pa·s/m^3)	Linear Decline ($\times 10^{12}$ Pa·s/m^3)
1	67.7	75.1
2	12.2	1.56
3	18.9	1.54
4	1.55	1.55

2.2. Simulation of The Valve Deformation and Flow Resistance

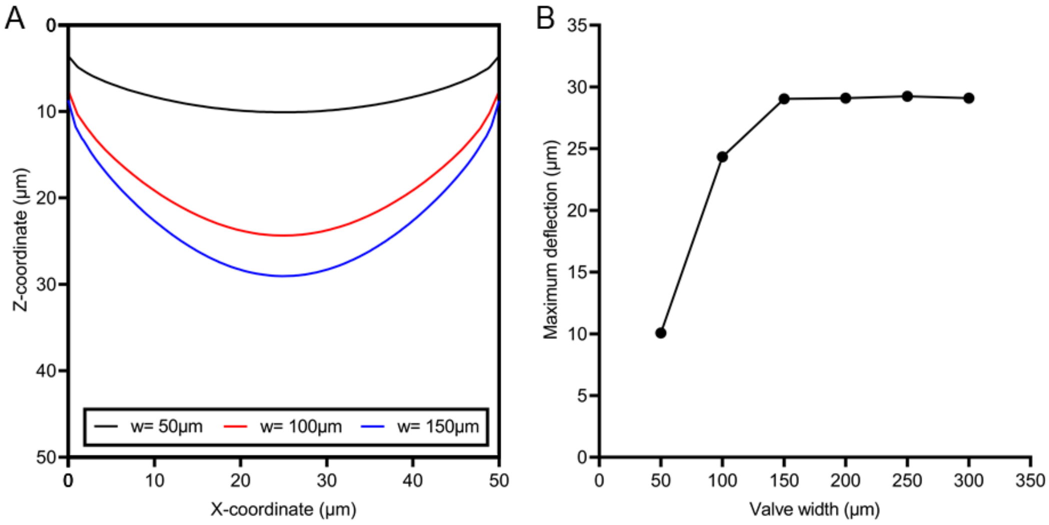

Figure S4. (**A**) The cross-sectional profile of the 20 μm-thick membrane deflected at 2000 mbar with varied valve width. (**B**) The maximum deflection of membranes with distinct valve widths when the 2000 mbar pressure applied.
